# Effect of Noise on Time-frequency Analysis of Vibrocardiographic Signals

**DOI:** 10.4172/2155-9538.1000202

**Published:** 2016-09-15

**Authors:** A Taebi, HA Mansy

**Affiliations:** Biomedical Acoustics Research Laboratory, University of Central Florida, United States

**Keywords:** Vibrocardiographic signal, Time-frequency analysis, Polynomial chirplet transform, Smoothed pseudo Wigner-Ville distribution, Noise

## Abstract

Recordings of biological signals such as vibrocardiography often contain contaminating noise. Noise sources may include respiratory, gastrointestinal, and muscles movement, or environmental noise. Depending on individual physiology and sensor location, the vibrocardiographic (VCG) signals may be obscured by these noises in the time-frequency plane, which may interfere with automated characterization of VCG. In this study, polynomial chirplet transform (PCT) and smoothed pseudo Wigner-Ville distribution (SPWVD) were used to estimate the instantaneous frequency (IF) of two simulated VCG signals. One simulated signal contained a time-varying IF while the other had a fixed IF. The error in estimating IF was then calculated for signal-to-noise ratios (SNR) from −10 to 10 dB. Analysis was repeated 100 times at each level of noise using randomized sets of white noise. Error analysis showed that the range of errors in estimating IF was wider when SNR decreased. Results also showed that PCT tended to outperform SPWVD at high SNR. For example, PCT was more accurate at SNR > 3 dB for a simulated VCG signal with constant frequency components, at SNR>−10 dB for a simulated VCG signal with time-varying frequency, and at SNR > 0 for an actual VCG.

## Introduction

Vibrocardiographic signals (VCG) are the vibrations induced by cardiac activity and measured at the chest surface. While early attempts of measuring VCG were performed in the late 1800 [1], several later studies [2–10] investigated VCG characteristics and their correlation with cardiac events and pathology. Time-frequency analysis (TFA) has been used to document temporal and spectral features of VCG [11,12] as well as other biomedical signals [13–19]. However, VCG signals are often contaminated with noise, which can be from physiological sources (e.g., respiration and muscle contraction), or external sources (e.g., building vibrations and instrument noise). Various methods have been utilized to remove noise from electrocardiographic and phonocardiography signals [20–24]. But, to the best of authors’ knowledge, there are no studies that focused on the analysis of VCG in the presence of noise. While a common objective of many signal analysis methods is to remove noise, addition of Gaussian noise assists data analysis in certain cases [25–30]. Noise removal is important as it may interfere with the operation of signal analysis methods. The current study aims at studying the effects of noise on the performance of two TFA methods that may be used for VCG analysis: polynomial chirplet transform (PCT) and smoothed pseudo Wigner-Ville distribution (SPWVD). A brief description of the calculated methods is given in the next section. Results are then presented and discussed, followed by conclusions in the last section.

## Materials and Methods

This section describes the simulated signals used and the methods of VCG data acquisition. The TFA performance evaluation method is also described.

### VCG signals and noise preparation

Two simulated VCGs were generated and added to background white noise with different SNR values. The general properties of the simulated signals are listed in Table 1.

Simulated VCG with constant frequencies: This signal consisted of two sinusoids with IF of 20 and 40 Hz, which can be described by

(1) $$x_{1}(t)=-A_{1}\text{sin}(2\pi (20)t+94)+0.9A_{1}\text{sin}(2\pi (40)t+188)$$

where the signal amplitude varied according to,

(2) $$A_{1}=\{\begin{array}{cc} 0 & 0<t\leq 0.25\\ 0.5-0.5\text{ cos}(2\pi (7t-5.25)) & 0.25<t\leq 0.40\\ 0 & 0.40<t\leq 0.70\\ 0.45-0.45\text{ cos}(2\pi (7t-5.25)) & 0.70<t\leq 0.85\\ 0 & 0.85<t\leq 1.00 \end{array}$$

Simulated VCG with varying frequency: This signal consisted of two sinusoids with constant and varying IFs, which can be described by

(3) $$x_{2}=A_{2}(y_{1}+y_{2})$$

where *y*_1_ and *y*_2_ were defined as,

(4) $$y_{1}=-0.5\text{ sin}(2\pi (40)t)$$

(5) $$y_{2}=\{\begin{array}{cc} 0 & 0<t\leq 0.25\\ z & 0.25<t\leq 0.40\\ 0 & 0.40<t\leq 0.70\\ z & 0.70<t\leq 0.85\\ 0 & 0.85<t\leq 1.00 \end{array}$$

where,

(6) $$z=\text{sin}(2\pi (870t^{2}-215t+20)t)0\leq t\leq 0.15$$

The signal amplitude also varied according to,

(7) $$A_{2}=\{\begin{array}{cc} 0 & 0<t\leq 0.25\\ 0.5-0.5\text{ cos}(2\pi (7t-5.25)) & 0.25<t\leq 0.40\\ 0 & 0.40<t\leq 0.70\\ 0.25-0.25\text{ cos}(2\pi (7t-5.25)) & 0.70<t\leq 0.85\\ 0 & 0.85<t\leq 1.00 \end{array}$$

Data acquisition of VCG: After IRB approval, a light-weight (2gm) accelerometer (PCB piezotronics, Depew, NY) was placed at the left sternal border and the 4^th^ intercostal space over the chest of healthy volunteers to measure the VCG signal. The signal was digitized at a rate of 3200 Hz and down-sampled to 320 Hz. Since respiratory noise have significant energy above 100 Hz [31], signals were filtered using a low-pass filter with a cut-off of 100 Hz to remove that noise. Math lab (R2015b, The Math Works, Inc, Natick, MA) was used to both acquire and process all signals.

**Figure 1:**
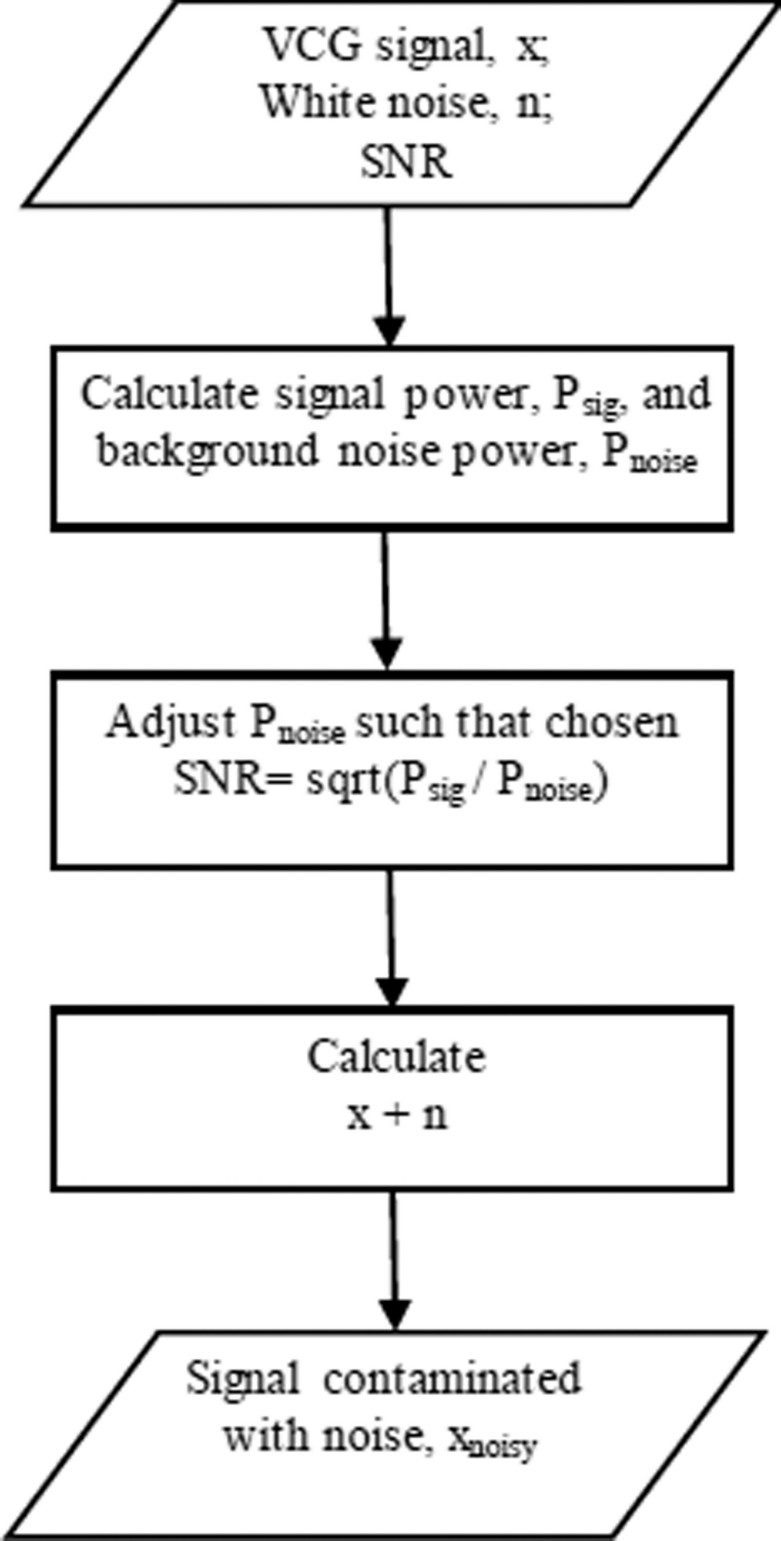
Block diagram describing the steps for generating the VCG signals contaminated with noise.

White noise: White noise with different levels of SNR ranging from −10 to 10 dB was generated and added to the simulated and actual VCG signals. The steps for generating the noise-added signals are shown in Figure 1.

### TFA techniques

Time-frequency distribution of the signals was estimated using two different TFA techniques; PCT and SPWVD. The theoretical details of these methods can be found elsewhere [32,33].

### Error analysis

The performance of each technique was assessed using the root-mean-square error (RMSE) between the signal actual and estimated IF values. The RMSE was calculated as:

(8) $$RMSE=\sqrt{\frac{\sum_{i=1}^{n}{\left(IF_{\text{act},i}-IF_{\text{est},i}\right)}^{2}}{n}}$$

where IF_act,i_ and IF_est,i_ are the actual and estimated IF at time i, respectively. RMSE values were normalized as:

(9) $$NRMSE=\frac{RMSE}{{\bar{IF}}_{\text{act}}}$$

where ${\bar{IF}}_{\text{act}}$ is the mean actual instantaneous frequency of each signal NRMSE was used in this study to measure the accuracy of TFA techniques in estimating IF, where lower NRMSE values would indicate higher accuracy.

For each SNR level, each signal was contaminated with 100 different white noise sets and analysis was performed for each case. Since the generated noise was different in each trial, analysis resulted in a range of errors (instead of one error value) at each SNR level. The results of the error analysis are presented in box-and-whisker plots in the Discussion section where the whisker ends represent the 1^st^ and 99^th^ percentiles. The error range was defined as the difference between these two percentiles. In addition, the Inter quartile range (IQR) was also calculated as the difference between the 75^th^ and 25^th^ percentiles.

## Results

**Figure 2:**
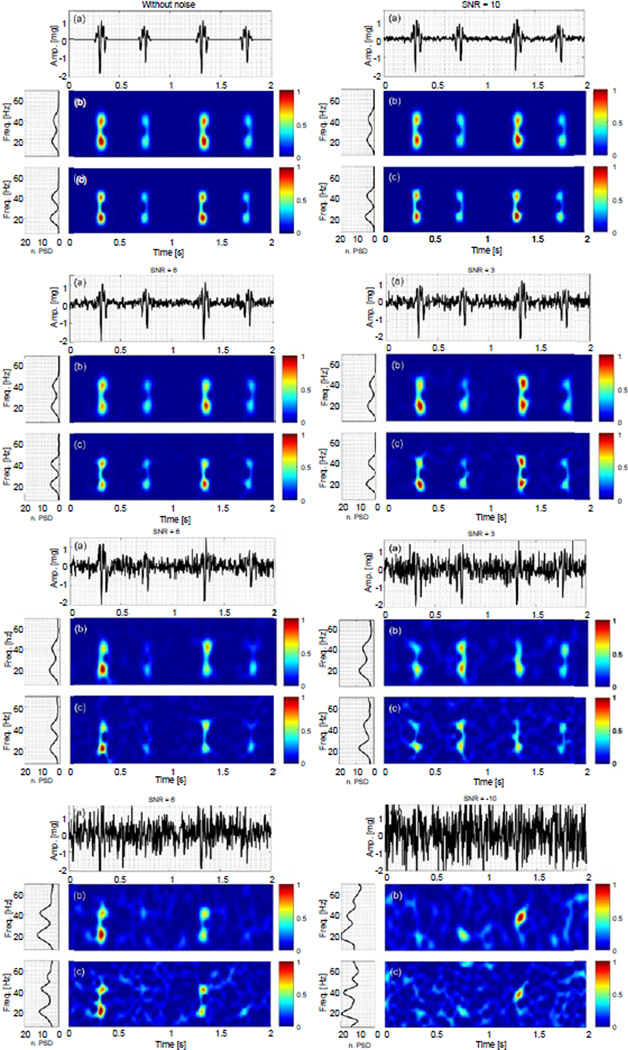
Simulated VCG with constant frequencies, x_1_, contaminated by white noise sets for −10 < SNR < 10 dB: (a) Time series. Time-frequency representation using (b) PCT, and (c) SPWVD, respectively.

**Figure 3:**
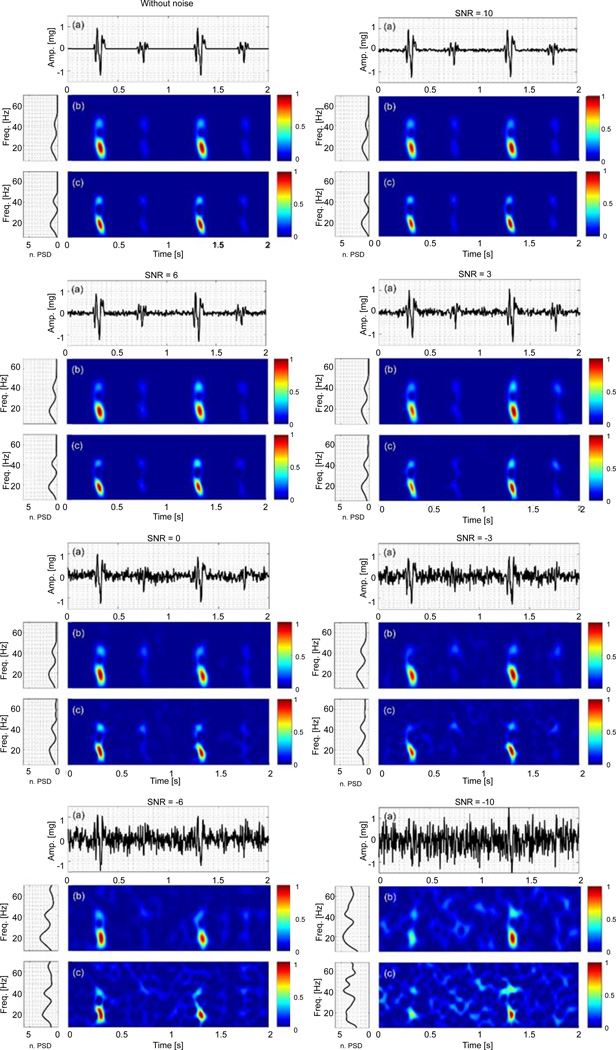
Simulated VCG with varying frequency, x_2_, contaminated by white noise sets for −10 < SNR < 10 dB: (a) Time series. Time-frequency representation using (b) PCT, and (c) SPWVD, respectively.

**Figure 4:**
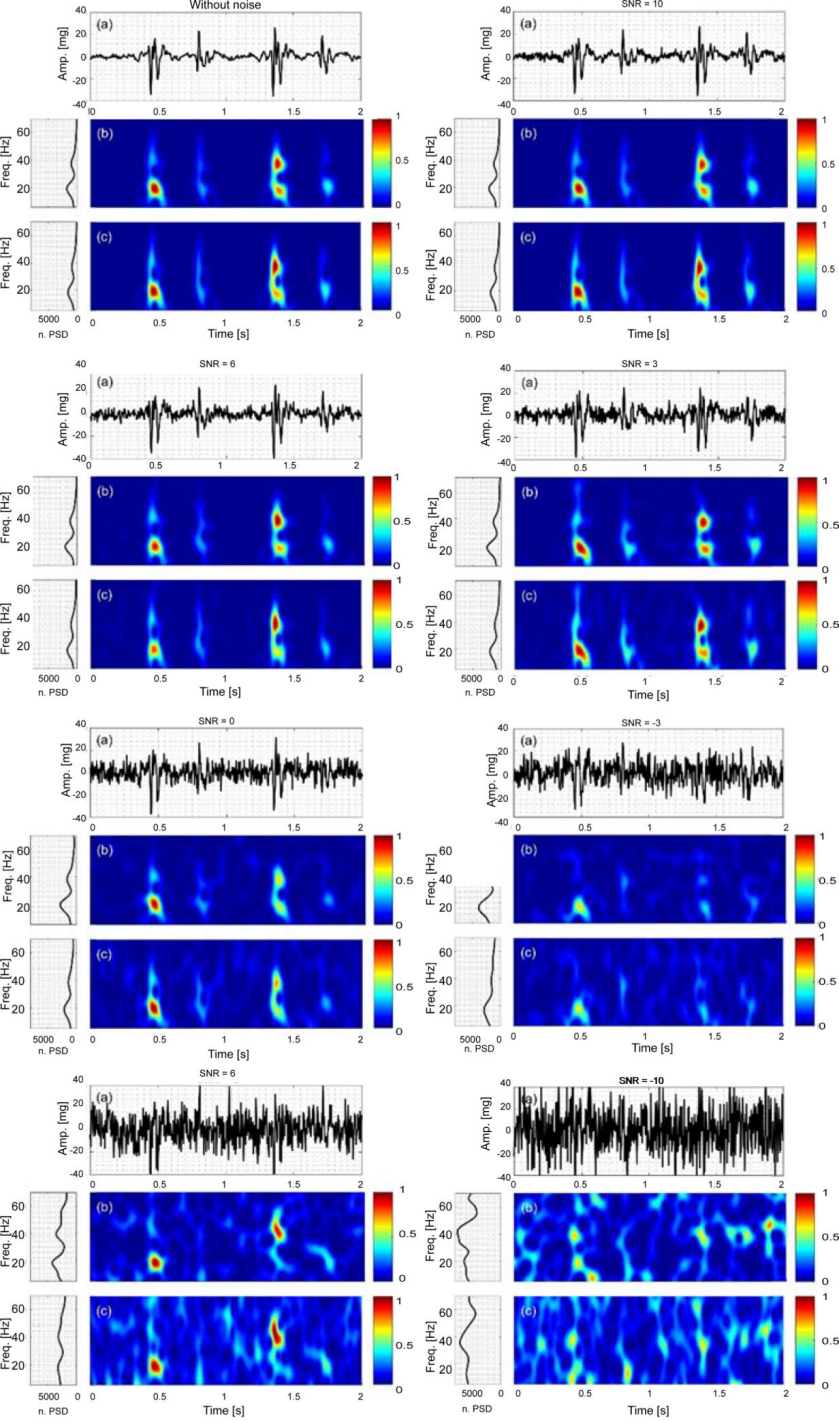
Actual VCG contaminated by white noise sets for −10 < SNR < 10 dB: (a) Time series. Time-frequency representation using (b) PCT, and (c) SPWVD, respectively.

Figures 2 and 3 show the time series, time-frequency representation and power spectral density (PSD) of the noise-added simulated signals. The PSD was calculated from the time-frequency representations, and normalized with respect to the signal energy. Since there is no significant energy seen above 70 Hz, the spectral information is only shown for frequencies up to this limit. The time-frequency representation and PSD of the actual VCG, which was polluted by white noise with different SNR values, were also estimated using the PCT and SPWVD, and shown in Figure 4. The figures show the data for only one noise set since the results for other noise sets were similar.

## Discussion

### Simulated VCG with constant frequencies

The first simulated VCG consisted of two constant frequency components. At each SNR, 100 different white noise sets were added to the signal, and the performance of TFA techniques in estimating the signal IF was assessed. Figure 2 shows that for the noise-added simulated VCGs at SNR > 3 dB, the time-frequency representation and the power spectral density did not appear significantly different from the ones for the signal without noise. For −3 < SNR < 3 dB, some extra energy peaks were seen in the time-frequency plane in addition to the signal actual frequency components. However, the PSD plots still showed two dominant peaks (representing two dominant frequency components at 20 and 40 Hz). At these SNRs, the signal frequency components appeared distorted. For example, at SNR = −3 dB, the TFA demonstrated some varying frequency behavior rather than a pure tone, which may lead to a misleading interpretation of the results. At SNR = −6 dB, PCT correctly depicted the two dominant frequencies of the signal, while the SPWVD showed an extra third peak at 55 Hz. The time-frequency representations of the signal were completely contaminated with noise at SNR = −10 dB, which resulted in power spectrums with more than 2 frequency peaks.

**Figure 5:**
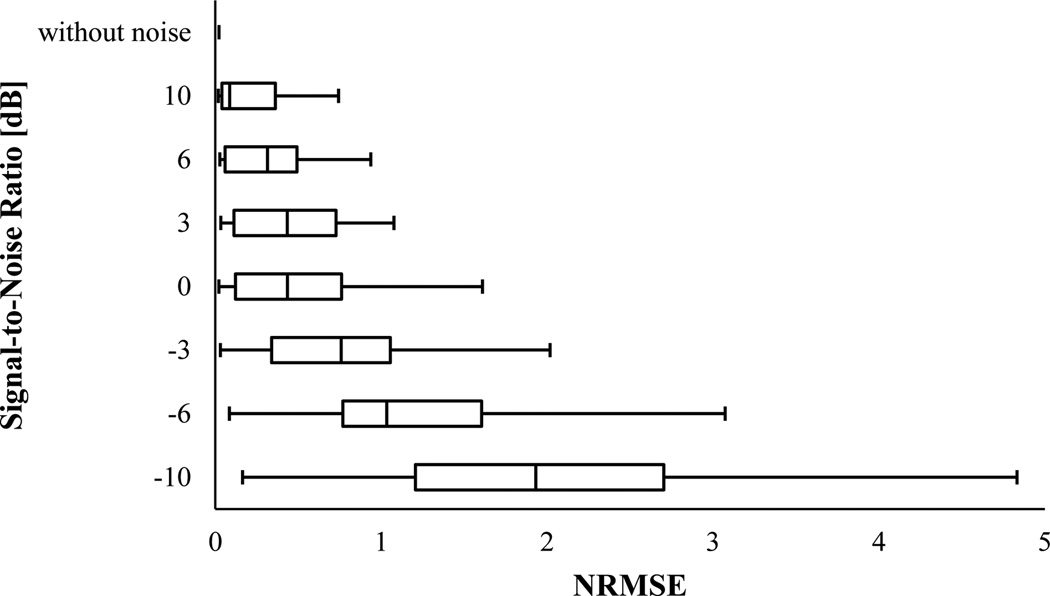
NRMSE in estimating IF of the simulated VCG with constant frequencies (x_1_) using PCT for different SNR. In this box-and-Whisker plot, the whisker ends represent the 1^st^ and 99^th^ percentiles.

**Figure 6:**
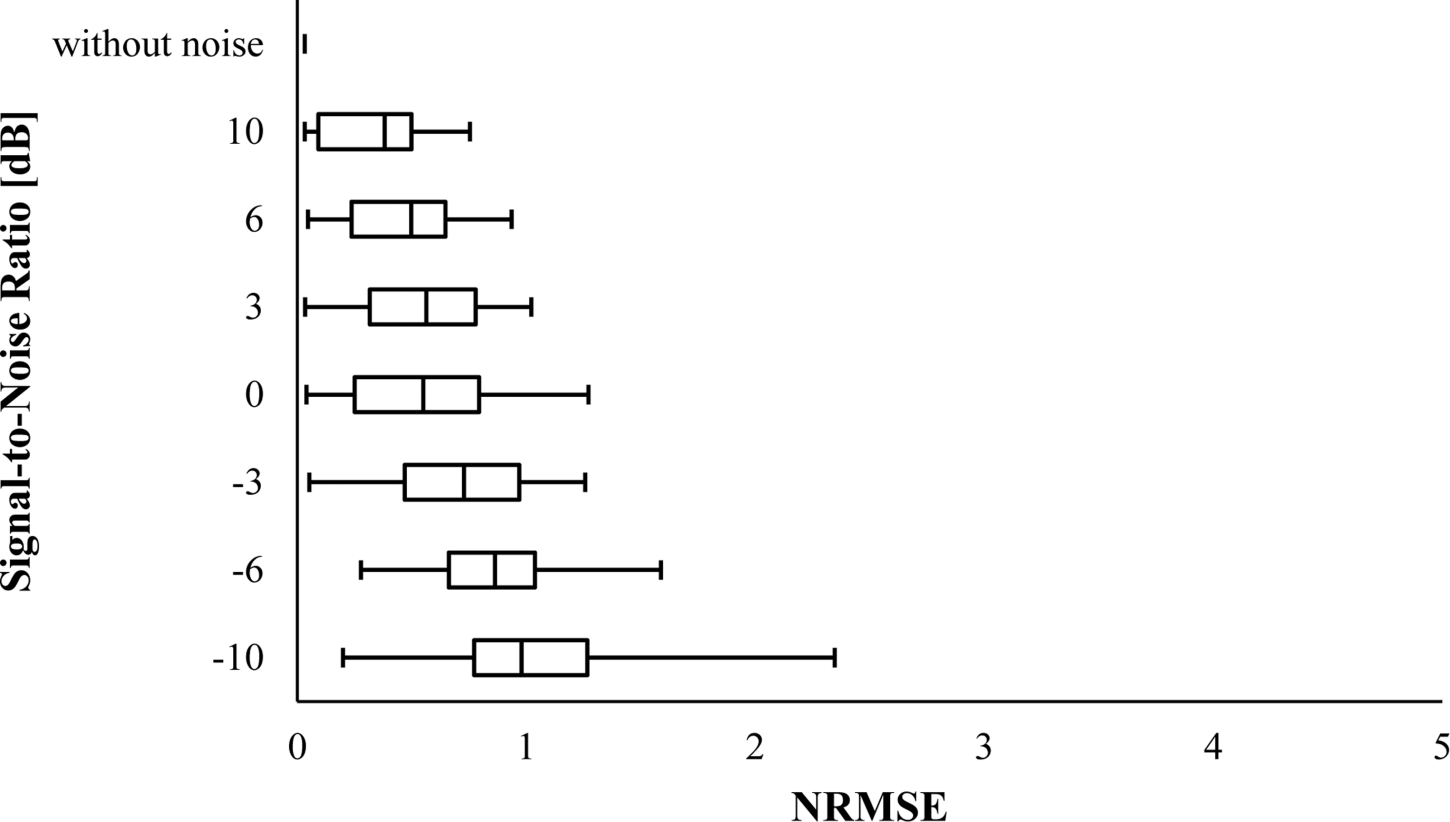
NRMSE in estimating IF of the simulated VCG with constant frequencies (x_1_) using SPWVD for different SNR. In this box-and-Whisker plot, the whisker ends represent the 1^st^ and 99^th^ percentiles.

Figures 5 and 6 show the NRMSE box-and-whisker plots for PCT and SPWVD, respectively. These plots suggested that PCT and SPWVD estimated the signal IF with higher NRMSE when the signal was polluted with noise. In general, the NRMSE medians and IQR increased as the SNR decreased for both PCT and SPWVD. The NRMSE median varied from 0.022 to 1.931 and from 0.032 to 0.979 for PCT and SPWVD, respectively, when SNR decreased from ∞ to −10 dB. At SNRs > 0 dB, PCT had lower NRMSE median and 75^th^ percentile compared to SPWVD. At lower SNR values (e.g. SNR = −3 and −6 dB), SPWVD estimated the signal IF with lower NRMSE median value and IQR. Therefore, for the simulated VCG with constant frequencies, one can conclude that PCT is more appropriate for IF estimation at higher SNRs, while SPWVD outperforms the PCT at lower SNR values.

### Simulated VCG with varying frequency

The second simulated VCG consisted of a constant and a varying frequency component. The TFA techniques were used to estimate the IF of the varying frequency component. At each SNR, the simulated signal was contaminated with 100 different white noise sets. Figure 3 shows that the time-frequency representations of the contaminated simulated VCGs with SNR > 3 dB were very similar to that without noise. For SNR ≤ 0 dB, the signal frequency components started to become distorted and extra energy peaks emerged in the time-frequency planes. These extra energy peaks were more clearly noticeable in the SPWVD than PCT. In addition, for SNR < −3 dB, a third power peak was shown up in the SPWVD PSD plot. Altogether, at lower SNR values, PCT provided time-frequency representations and PSD plots that are lesser affected by the presence of white noise than SPWVD.

**Figure 7:**
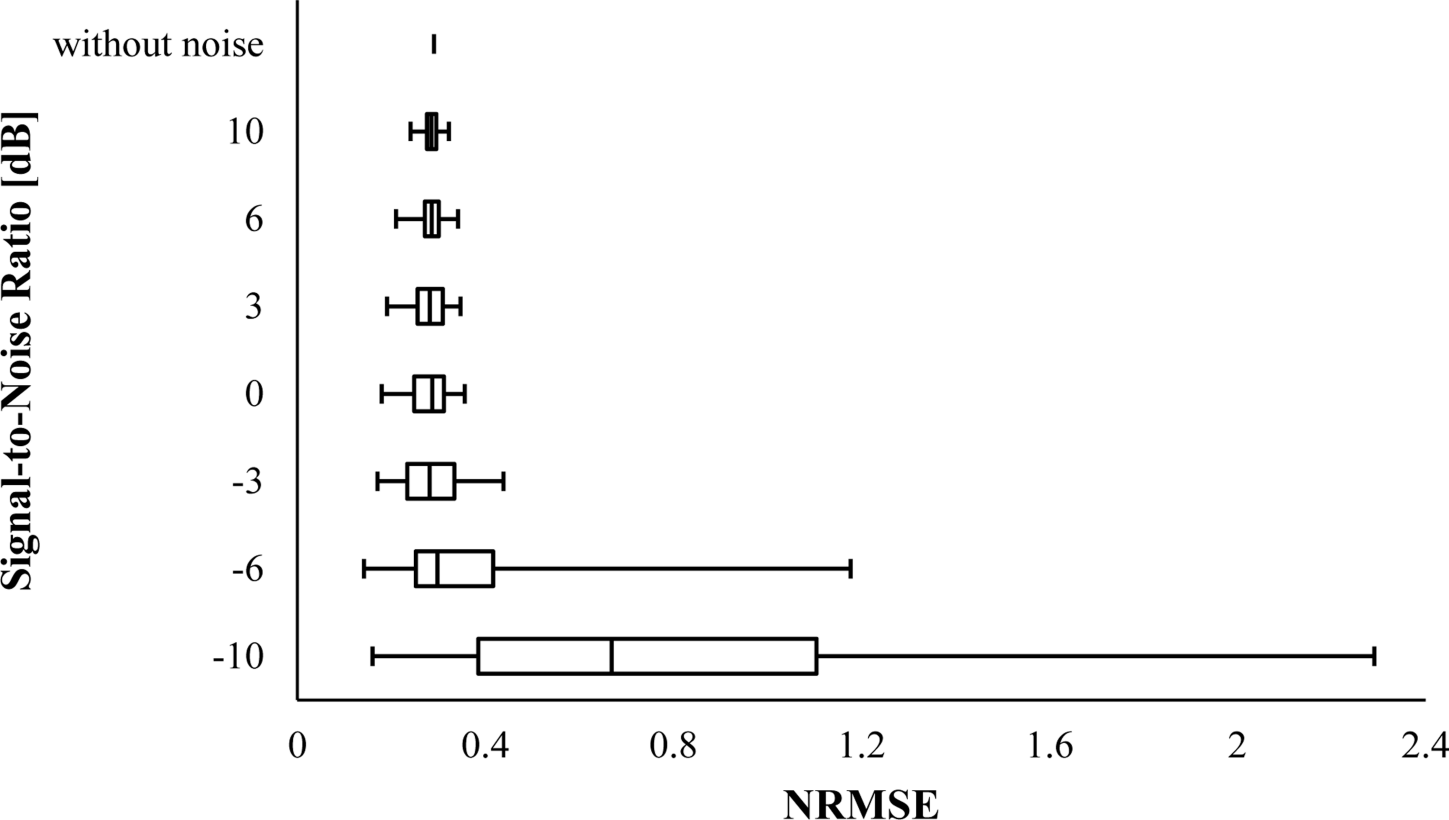
NRMSE in estimating IF of the synthetic VCG with varying frequency (x_2_) using PCT for different SNR. In this box-and-Whisker plot, the whisker ends represent the 1^st^ and 99^th^ percentiles.

**Figure 8:**
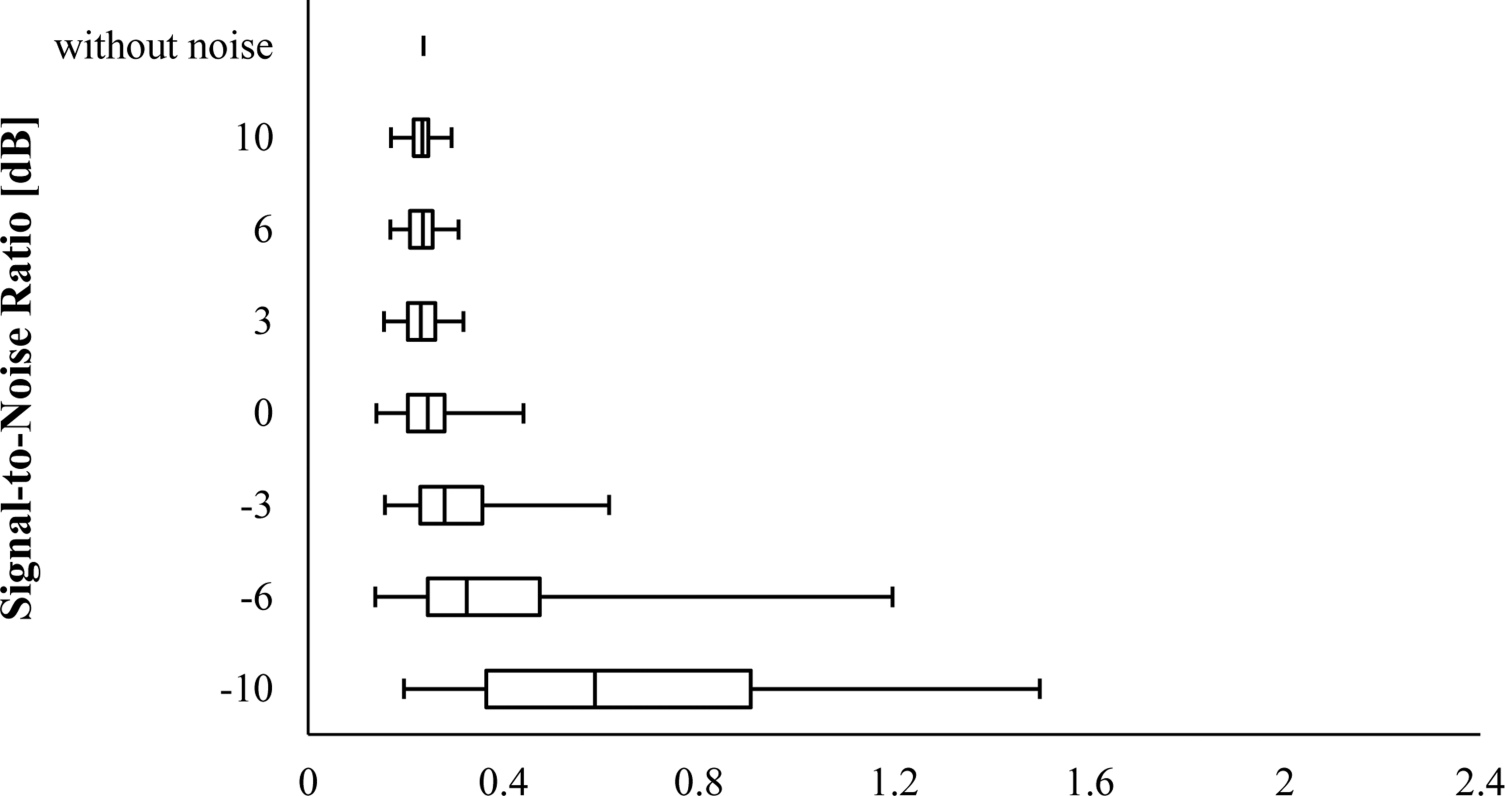
NRMSE in estimating IF of the synthetic VCG with varying frequency (x_2_) using SPWVD for different SNR. In this box-and-Whisker plot, the whisker ends represent the 1^st^ and 99^th^ percentiles.

The results of the IF error analysis of the simulated VCG with varying frequency were described as box-and-whisker plots in Figures 7 and 8. For SNR ≥ −6 dB, PCT had almost the same NRMSE median (~ 0.285) as that without noise, however, both NRMSE IQR and range increased as SNR decreased. At SNR = −10 dB, the NRMSE median drastically increased to ~ 0.668. SPWVD also estimated the IF with an almost unvaried median NRMSE value of 0.235 at SNR ≥ 0 dB, which was very close to NRMSE for the case without noise. But, for SNRs smaller than 0 dB, the IF estimation error of SPWVD rose to about 0.587. For both PCT and SPWVD, smaller SNR value leads to a higher NRMSE IQR. Altogether, SPWVD consistently had lower NRMSE median (~ 0.235 vs ~ 0.285) and higher NRMSE IQR and range for SNR ≥ −6 dB. Considering that a lower uncertainty in IF estimation may be desirable, one can conclude that PCT may be preferred over SPWVD for estimating IF of a noisy signal. In summary, the error analysis for the simulated signals suggests that:

- For VCG signals with time-varying frequency components, PCT may provide more accurate estimation of IF in the presence of white noise.
- For VCG signals with time-independent frequency components, PCT may give more accurate IF estimations when SNR > 3 dB.

### Actual VCG in noise

The actual VCG was also contaminated by white noise with different SNRs. Figure 4 shows the time series, time-frequency representations and PSD plots of the actual VCG at different SNR values. For higher SNR values (e.g. SNR ≥ 6 dB), the time-frequency representation and PSD of the signal were not significantly affected by the white noise presence. For SNR < 6 dB, the time-frequency representation started to become distorted. For instance, at SNR = −3 dB, the VCG1 higher frequency component started to disappear from the time-frequency representation. At lower SNRs, more extra energy peaks were shown up in the time-frequency plane. For example, the PSD of the actual VCG without noise had 2 peaks, however, the PSD graph had more than 2 peaks for small SNRs (e.g. SNR = −6 dB).

Since the actual IF of the VCG signal was not known, it was not possible to perform a same exact error analysis that had been done for the simulated VCGs. Instead, the performance of TFA techniques in estimating the VCG IF was evaluated using the following measure:

(10) $$NRMSE_{VCG}=\sqrt{\frac{\sum_{i=1}^{n}{\left(IF_{\infty ,i}-IF_{\text{noisy},i}\right)}^{2}}{n}}/{\bar{IF}}_{\infty }$$

**Figure 9:**
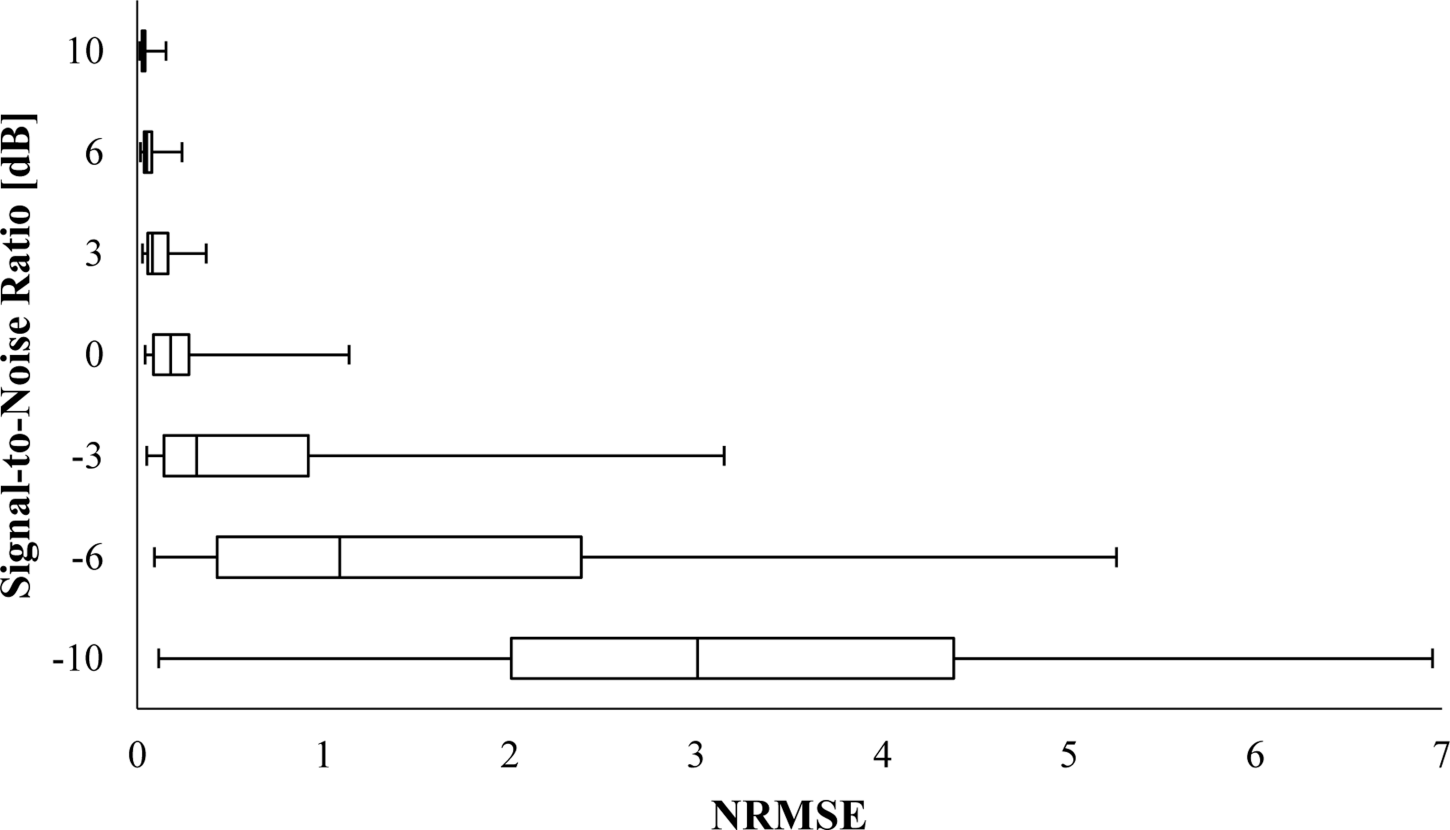
NRMSE in estimating IF of the actual VCG using PCT for different SNR. In this box-and-Whisker plot, the whisker ends represent the 1^st^ and 99^th^ percentiles.

**Figure 10:**
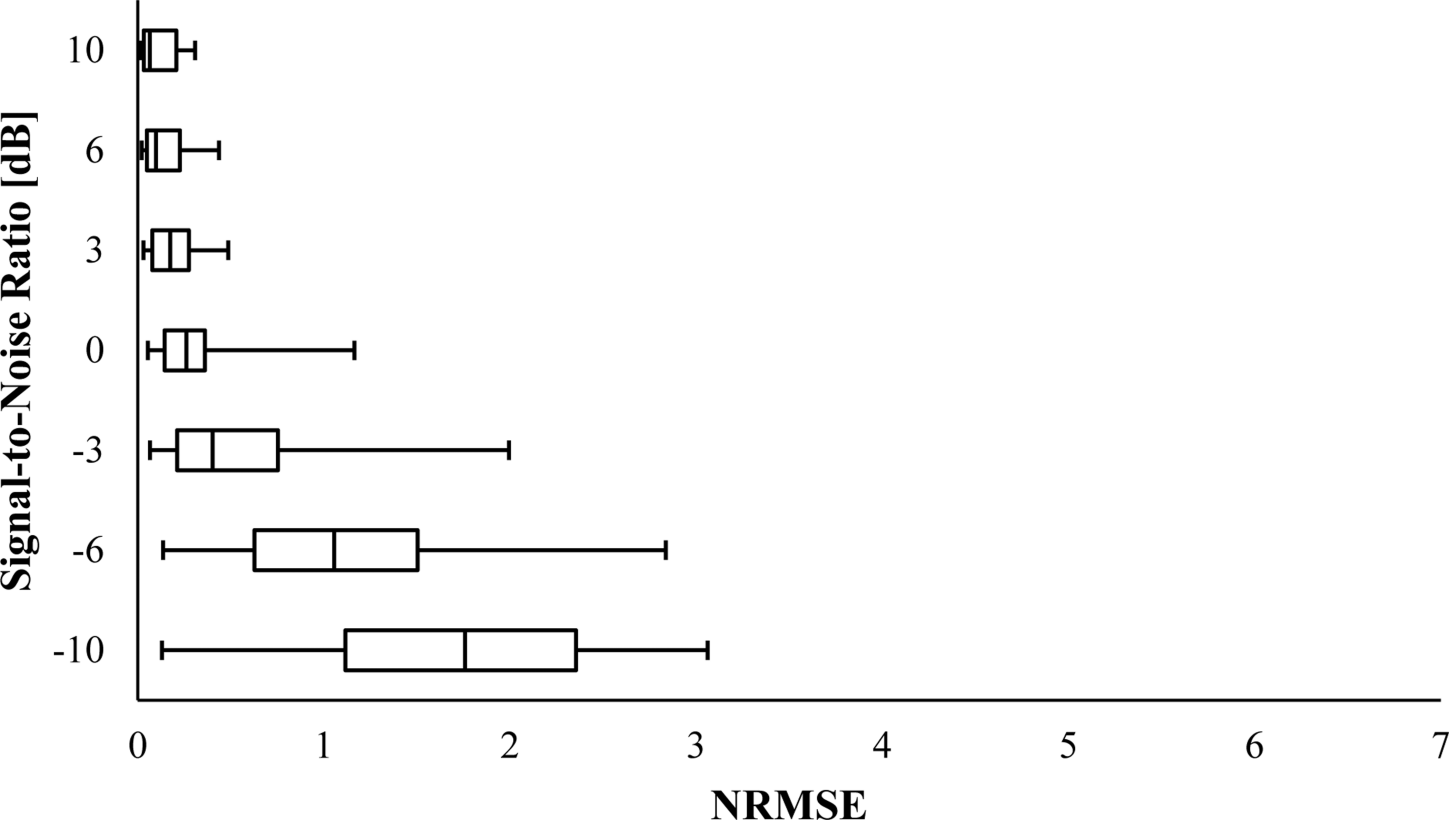
NRMSE in estimating IF of the actual VCG using SPWVD for different SNR. In this box-and-Whisker plot, the whisker ends represent the 1^st^ and 99^th^ percentiles.

Where IF_∞,i_ and IF_noisy,i_ were the estimated IF of the VCG without noise and the noise-added VCG at time i, respectively; and ${\bar{IF}}_{\infty }$ was mean of the estimated IF of the signal without noise. The actual VCG was polluted by 100 different white Gaussian noise sets at each SNR value. Then, Eq. 10 was used to find the error in estimating IF. The error analysis results are presented in Figures 9 and 10. It can be seen that PCT estimated the signal IF with lower NRMSE median and IQR at SNR ≥ 0 dB. The median, IQR and range of NRMSE values are listed in Table 2 for the simulated and actual VCGs at signal-to-noise ratios from −10 to 10 dB.

## Conclusion

The goal of this study was to compare the ability of the polynomial chirplet transform and smoothed pseudo Wigner-Ville distribution in providing accurate time-frequency estimates for VCG signals contaminated by white noise. The accuracy of the different methods in determining the IF was tested using two simulated VCG signals. The estimated and actual signal IF were compared. Results suggest that at high SNRs, PCT was more accurate than SPWVD in estimating the frequency components of a signal with time-independent IF. For a signal with varying frequency components, SPWVD resulted in a smaller median error but larger error range than PCT. Since lower error range (i.e. lower uncertainty) may be desirable, PCT may be chosen over SPWVD in estimating the IF of a VCG with time-varying frequency components. More studies may be warranted to document the time-frequency characteristics of VCG signals in health and disease.

## Figures and Tables

**Table 1:** General properties of the simulated signals used in the current study.

Signal description	Peak to peak amplitude	Signal length above 5% of peak to peak amplitude (ms)	Frequency or Frequency range (Hz)
Simulated VCG with constant freq., *x*_1_	2.8	112	20 and 40
Simulated VCG with varying freq., *x*_2_	2.4	112	7 to 20, and 40

**Table 2:** A summary of NRMSE analysis of simulated and actual VCG signals for different SNR. The NRMSE values are reported as: Median (IQR, Range). Here, the range is defined as the difference between 1^st^ and 99^th^ percentiles.

	Signal-to-Noise Ratio (dB)								
		−10	−6	−3	0	3	6	10	without noise
**Simulated VCG** **with constant** **frequencies**	**PCT**	1.93 (1.50, 4.67)	1.03 (0.84, 2.99)	0.76 (0.72, 1.99)	0.43 (0.64, 1.59)	0.43 (0.61, 1.04)	0.31 (0.43, 0.91)	0.09 (0.32, 0.72)	0.02 (0.00, 0.00)
	**SPWVD**	0.98 (0.49, 2.15)	0.86 (0.38, 1.31)	0.73 (0.50, 1.21)	0.55 (0.54, 1.23)	0.56 (0.46, 0.99)	0.50 (0.41, 0.89)	0.38 (0.41, 0.72)	0.03 (0.00, 0.00)
**Simulated VCG** **with varying** **frequency**	**PCT**	0.67 (0.72, 2.13)	0.30 (0.16, 1.03)	0.28 (0.10, 0.27)	0.29 (0.06, 0.18)	0.28 (0.05, 0.16)	0.28 (0.03, 0.13)	0.28 (0.02, 0.08)	0.29 (0.00, 0.00)
	**SPWVD**	0.59 (0.54, 1.30)	0.32 (0.23, 1.06)	0.28 (0.13, 0.46)	0.24 (0.08, 0.30)	0.23 (0.06, 0.16)	0.23 (0.05, 0.14)	0.23 (0.03, 0.12)	0.23 (0.00, 0.00)
**Actual** **VCG**	**PCT**	3.00 (2.37, 6.83)	1.09 (1.95, 5.16)	0.32 (0.77, 3.10)	0.18 (0.19, 1.09)	0.08 (0.11, 0.34)	0.05 (0.04, 0.22)	0.03 (0.01, 0.14)	N/A
	**SPWVD**	1.76 (1.24, 2.93)	1.05 (0.88, 2.70)	0.40 (0.54, 1.93)	0.26 (0.22, 1.11)	0.17 (0.19, 0.45)	0.10 (0.18, 0.41)	0.06 (0.18, 0.29)	N/A
